# Forecasting the nearly unforecastable: why aren’t airline bookings adhering to the prediction algorithm?

**DOI:** 10.1007/s10660-021-09457-0

**Published:** 2021-01-13

**Authors:** Saravanan Thirumuruganathan, Soon-gyo Jung, Dianne Ramirez Robillos, Joni Salminen, Bernard J. Jansen

**Affiliations:** 1 Qatar Computing Research Institute, Hamad Bin Khalifa University, Education City, Qatar; 2 School of Statistics, University of the Philippines, Diliman, Philippines

**Keywords:** Prediction, Recommendation, Airlines, Travel, User evaluation

## Abstract

Using 27 million flight bookings for 2 years from a major international airline company, we built a Next Likely Destination model to ascertain customers’ next flight booking. The resulting model achieves an 89% predictive accuracy using historical data. A unique aspect of the model is the incorporation of self-competence, where the model defers when it cannot reasonably make a recommendation. We then compare the performance of the Next Likely Destination model in a real-life consumer study with 35,000 actual airline customers. In the user study, the model obtains a 51% predictive accuracy. What happened? The Individual Behavior Framework theory provides insights into possibly explaining this inconsistency in evaluation outcomes. Research results indicate that algorithmic approaches in competitive industries must account for shifting customer preferences, changes to the travel environment, and confounding business effects rather than relying solely on historical data.

## Introduction

Prediction algorithms are integral for matching users with relevant products, usually by leveraging historical user information [3, 34]. A related situation is not stringently predicting the future product but recommending a product that does not occur in historical data but that the customer might like. These recommendation methodologies characteristically focus on novelty at an appropriate place in a sequence [1].

There are situations, however, where the goal is to both predict and/or recommend. Flight booking is one such situation because destination patterns for a given customer could remain stable or change for a host of confounding factors [23, 58]. This challenging flight booking domain is the focus of this research, with a specific interest in forecasting customer behaviors for this blended prediction-recommendation context. Our research goal is to *investigate the effectiveness of algorithmic approaches in suggesting the next destination booking for an airline customer.* Most of the prior work in both the prediction and recommendation areas has been developed and evaluated solely on historical datasets. Few prior studies have evaluated these developed models with real customers [40]. We do assessments with both historical data and real customers in this research. We develop the next likely destination (NLD) model, evaluate the NLD model using historical data, and test the model with real customers in an operational business environment. Thus, we can compare the ‘lab’ and ‘real’ performances of the model.

While there has been extensive work on recommendations for virtual goods [24] (such as movies, songs, or news articles), there has been a relative paucity of research and evaluation on recommenders for physical services. As we describe later, recommending the next booking destination is complicated and influenced by a number of factors. For example, a city that might be an apt recommendation during the summer might be inappropriate in winter. Even more challenging issues are the operational constraints inherent in the airline industry, such as booking windows, that are completely elided, to our knowledge, in prior work on recommendations systems. Furthermore, prior work often treats all customers equivalently or at least segments customers into broad segments [11, 29, 53]. However, for an airline, some customers provide more revenue than others. Hence, it is preferential to be more accurate for these high-value customers. To the best of our knowledge, there is no prior work on ND recommendations that factor in the practical operational and revenue considerations, although the revenues aspects are of increasing concern [65].

The NLD model has a novel feature combination of (a) temporal, (b) geographic, (c) self-competence, and (d) revenue aspects. To the best of our knowledge, prior work has not previously studied the next destination (ND) problem in-depth with the combined range of feature constraints employed in this research, nor has it been constrained by actual operational business aspects, such as booking windows, as we are in this research. Additionally, to our knowledge, prior work has not reported detailed evaluations using both historical data and real customers as we do in this research. As such, the research reported here is novel.

In this research, we collaborate with one of the world’s largest and most highly rated airlines to investigate the ND booking issue in the airline industry. From a business perspective, the ND situation aims to identify the most likely next booking destination of a customer during a given booking window. In the furiously competitive market, the company’s business objective is to use the NLD model to increase the booking window (i.e., get the customer to book the flight earlier) to lock in the customer’s business.

To investigate this ND opportunity thoroughly, we first pilot test a naive implementation to evaluate the effectiveness of recommending the ND. Based on modest results, we enhance the sophistication of our approach, developing the NLD model. We then conduct an evaluation [36] of the NLD model using historical data. We then test the NLD model using 35,000 actual customers of the airline company.

Results show substantially different ND accuracy results for the NLD model using historical data versus actual customers. The implications are that these recommendation models, in the context of flight bookings at least, require validation from actual customers. Results show that one cannot rely solely on historical data to get an accurate measure of the algorithmic effectiveness and that approaches must be integrated thoroughly with the overall customer relations efforts to achieve comprehensive business objectives. Additionally, algorithmic methods in the travel domain must be resilient to black swan events [77] and crises affecting travel (e.g., COVID-19). We discuss the implications for deploying such systems in these complex domains via the Individual Behavior Framework theory.

## Prior work

By analyzing past behavior to build a profile of interests [62], recommendation approaches provide suggestions that may interest customers. The recommendation model then leverages this profile to recommend potential future destinations [80]. Prior work, such as Quadrana, Cremonesi, and Jannach [63], presents an overview of sequence-based recommendations. Generally, such approaches can be accurate if the task is one of algorithmic prediction [21]. A known limitation of this approach, which applies to our context when deployed with users, is that the suggestions may be states that are nearly identical to what the user knows already.

There are contexts in which one may want to serendipitously recommend new items that the user probably will like but may not be aware of based on associated interests and external events [16, 50]. With limited exceptions [6, 88], there has been sparse research in the serendipity area with large-scale datasets. This context is especially applicable to the domain of flight bookings, which is both prediction (i.e., going to a prior destination) and recommendation (i.e., going to a new destination), combined with continual upheaval in how potential travelers make bookings [47]. Prior research using historical data investigated e-commerce recommendations [31] and reported that various factors [5] should be considered in tandem with recommendations [10]. However, the researchers [31] did not have access to actual revenue data or the specific deployed algorithm. These confounding factors raise concerns about using historical data to evaluate algorithmic accuracy.

Previous work does include the discovery of similar customers based on their temporal histories or demographics [20]. There have been various algorithmic approaches employed in this regard [60, 80, 88] using both aggregated data and individual user data [42]. Findings show that combining multiple methods is not always more effective than a single method, and in given temporal contexts, different approaches perform differently [80]. In the competitive airline industry, customer prediction and recommendation are active avenues of pursuit aimed at discerning customer behaviors [27], generating revenue [64], endearing customer loyalty, and enhancing customer experience [12]. In sum, predicting the behavior of airline travelers is challenging due to the confounding factors, including individual travel attributes, destinations, and the situation of the market [48].

There has been considerable interest in recommender systems for travel [74], and we leverage prior work for the ND problem that studied the impact of item dependencies [7, 26]. The ND problem is especially difficult in the airline domain, where selecting a destination can be impacted by various confounding factors [4, 25, 61]. Other novel aspects of travel recommendation involve the relationship between location and time, as examined in some prior research [22, 85]. Pan, MacLaurin, and Crotts [59] seek to improve the forecasting accuracy of demand using external search engine data Addressing these issues is crucial, as consumers exploit dynamic online pricing information [47] and exhibit strategic purchasing behavior [49], both of which are impactful for the competitive airline industry. As the mentioned work reports, predicting airline travel is a task riddled with confounding factors, including airline competition at airports, cost-conscious travelers, and multiple choices in destinations [41].

Despite this work, there have been few online evaluations of reported recommendation systems [88] with real customers. One exception is [35], which used rating data provided by previous customers of Booking.com and implemented three methods to compare them to the Booking.com baseline. The authors conducted an online A/B test with live users, and the NB-based recommender increased user engagement. However, the authors did not report if actual bookings increased. This is an aspect that we address in the findings of our research, with surprising results that perhaps shed light on why so few user studies [82] are actually reported in the literature.

In summary, we are investigating a specific ND task with nuanced properties that both build on and differ from prior research. Due to these challenges and despite the reported success in online retailing, recommender systems have been less prevalent in flight itinerary selection processes [56]. Table 1 outlines the challenging aspects of the ND problem, with difficulties for recommender systems in nearly every area.

**Table 1:** Challenges inherent in the next destination (ND) issue

Optimization constraints	Prior work	ND issue
Goal	Prior work has generally focused on either prediction or recommendation and seldom blended the two [87]	Mixture of prediction and recommendation
Interplay of constraints (e.g., timing, location, cost, external events, preferences, revenue, booking window, customer value)	Prior work has typically focused on one (e.g., location) or two (e.g., location and time) restraining attributes [38]	Presence of multiple restraining attributes interacting to determine the ND booking decision
Business value	Has rarely been examined or considered in prior work [35]	Critical aspect of the ND problem
Data sparsity	Has been extensively acknowledged and examined in prior work [11, 28]	Critical aspect of the ND problem
Cold start	Has been extensively acknowledged and examined in prior work [45, 89]	Critical aspect of the ND problem
Changing characteristics of customers and/or the environment	Models in prior work have mostly been trained and tested using historical data, thereby ignoring changes in customers and the environment [68, 75]	To be implemented on actual customers of a major international airline
Total set of constraints	To the best of our knowledge, no prior work has simultaneously addressed all of the constraints of the ND problem	ND issue is a complex real-world challenge faced by the airline and similar businesses

There are still several unanswered questions in the ND context. *What algorithmic model can best address the ND issue? How does algorithmic accuracy of historical data compare to that with accuracy using real people? How do recommendation algorithms perform with actual customers for flight bookings? How do these algorithms perform within the overall business context?* These are questions that motivate our research.

## Research objectives

Our research objectives are:

- *Develop an effective ND recommendation model for flight bookings for airline customers*;
  Although there have been studies of flight recommendations [17] based on user preferences [79], predicting the next flight booking has, to our knowledge, not been addressed in the prior work.
- *Evaluate the accuracy of the developed ND model employing historical flight booking data*;
  Typically, recommendation approaches in the travel domain are evaluated using historical data [48] using a single metric, such as accuracy [9, 55]. In this research, we present our evaluation with several metrics (e.g., accuracy at different top destinations, high- versus low-value customers, seasonality, etc.) for a more robust evaluation.
- *Test the accuracy of the developed ND model using actual airline customers in a real-world context*.
  There has been scant prior work in the actual evaluation of travel recommendation or prediction algorithms, with those that do report some evaluation relying on historical data or some proxy [79]. We could locate only one study that actually evaluated the recommendations in a real-life setting [35], and this one study did not employ actual airline customers but rather website visitors. Thus, there is little research on the actual deployment of these travel recommendation systems, raising questions concerning if these approaches work in real situations [54]. As such, this evaluation with actual airline customers is a novel aspect of the research.

The research problem is highly impactful, and it has practical value. In support of a customer retention program, the airline company wants to analyze historical flight booking data from passengers to identify an ND. The company then sends potential customers an online offer to incentivize them to book a flight for this destination, with the business goal of locking in the booking and increasing the booking window. This increased booking window reduces the chance of the customers booking the flight with a competing airline.

## Methodology

### Data collection and preparation

Our data collection site is an international airline with more than 165 destinations in dozens of countries. Our dataset is 27 million flight bookings from nearly 20 million customers during the 2016–2018 period. All personally identifying variables were masked in the dataset and not available to researchers. Specifically, the dataset contains the following information:

- *Customer details* Customer ID, frequent flier number, frequent flier level (0—not a member to 5—highest level), gender, and nationality.
- *Trip details* Booking reference code (identifier for the booking), booking date, booking channel used in creating the bookings (group, online, or other), point of sale city (where the ticket was purchased), flight number, flight date, origin and destination cities (airport codes), and cabin class (first, business, economy).
- *Flight information* For each sector (i.e., origin city–destination city), we collected the departure time in GMT, the aircraft type, and the duration of the flight in minutes. This allowed us to factor in the effects of departure time, aircraft preferences, and impact of flight interval.
- *Additional information* We also garnered touristic metadata about each city served by the airline. These include geographic details (e.g., continent, weather), type of tourism served (e.g., adventure, historical), the best time to visit, etc.

### NLD model development challenges

Designing an effective NLD model requires overcoming several specific algorithmic and operational issues.

### Algorithmic challenges

The major algorithmic challenges are:

- *Contextual and temporal* A suitable NLD model must take into account both context and time, as the destination and time are intrinsically connected. For example, a customer’s hometown might be an accurate ND during Christmas but not in the summer.
- *Sparsity and skewness* The user-item interaction matrix is sparse along multiple dimensions. In analyzing our dataset of 27 million trips made by almost 20 million customers, we find that nearly 90% of the customers take fewer than three yearly trips, and most customers travel to a small set of destinations.
- *Incomplete and partial* The airline industry is highly competitive, where most customers are price-sensitive and use price comparison sites to find the cheapest airlines. This airline hopping accentuates the sparsity issue and results in incomplete customer information.

### Operational challenges

While traditional accuracy metrics are important, maximizing the business value for the company in the ND situation is much more important.

- *Domain knowledge* The airline’s customer retention, data analytics, and revenue management team have developed domain expertise for which the NLD model must account. For example, certain flights at certain periods might have historical booking levels that make that destination unattractive, referred to as load factors in the airline domain.
- *Priority passengers* There is a fundamental tension between the traditional optimization objective of recommender systems (*maximize accuracy*) and that of ND (*maximize business value*). In a practical ND setting, not all customers are equal; some generate more revenue for the company than others. Learning the latent space of the former is more important.
- *Self-competence* Typical recommendation algorithms predict the rating of a product for all users. However, such an approach is not appropriate for the NLD model. Since there is a monetary cost for incorrect ND predictions, it is important that the NLD model make a prediction only when the recommender is certain. Therefore, the NLD Model can choose to abstain if it is not confident [83]. This situation is analogous to a human expert saying, “I do not know,” which is often preferable to making incorrect guesses.

### Pilot test

**Fig. 1:**
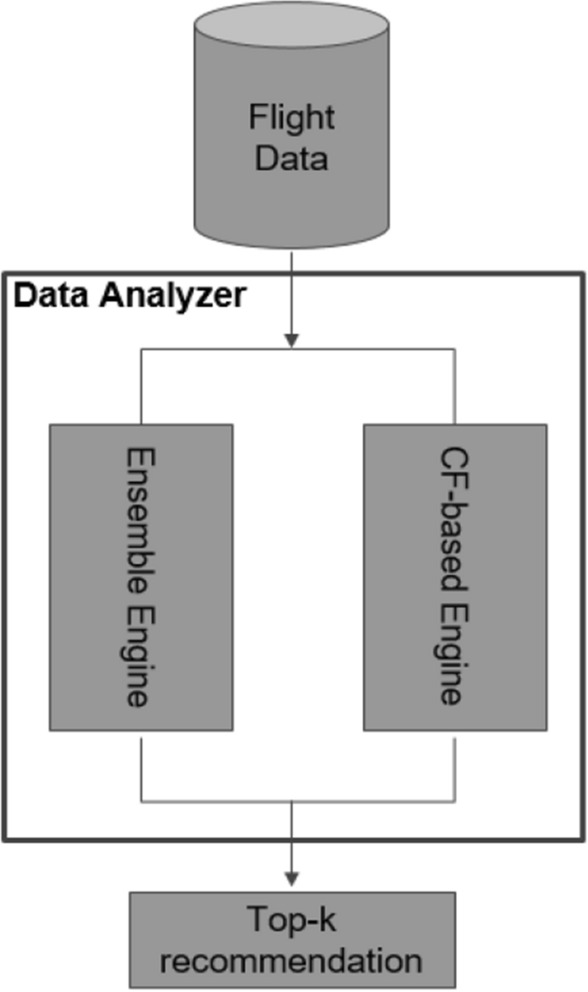
Methodological framework for the ND pilot model

Given these robust challenges, we determined that the best course of action was to conduct a pilot test, reported in [33], as a proof of concept by applying both an ensemble method and a collaborative filtering method, as shown in Fig. 1.

For each customer, we selected the top five destinations that our algorithms predicted the customer would book next or would like to book, based on the customer’s historical booking pattern (i.e., precision at 5 (P@5)). For the performance comparison, we used a baseline of recommending the most popular destinations (i.e., using no algorithm but recommending the most popular destinations). The baseline has an accuracy P@5 of 19.0%. After training our models using historical data, we tested the model on a set of approximately 100,000 actual airline customer records, generating ND recommendations for each.

The ensemble method merges several classifiers to achieve better performance than any single classifier [46]. The second approach was collaborative filtering, which finds users similar to a given user and then recommends these similar users’ recent destinations to that user [71]. Based on historical data, the ensemble method had an accuracy of 47.6% (148% better than baseline), and collaborative filtering had an accuracy of 24.8% (30% better than the baseline).

We then randomly selected 10,000 customers for an actual customer test, splitting the customers into groups of 4500 for the ensemble recommendation, 4500 for the collaborative filtering recommendation, and 1000 for a control group. The test groups were sent marketing messages crafted by the airline company’s marketing department, and the control group was sent no marketing messages. Each marketing message contained a recommendation for one of the selected destinations and offered bonus miles for booking a flight to one of the destinations within the offer window. Our combined algorithmic approaches resulted in prediction accuracy during the customer testing of approximately 23%, as measured by the customers selecting the predicted destinations. Results showed a 16% increase in bookings of the test groups compared to those of the control group. The overall predictive power was 23%, with collaborative filtering having a predictive power of 30% and 19% for the ensemble method. The results from both the historical data and user study prompted us to pursue a more sophisticated approach.

### NLD model development

Our early experiment addressed above led us to believe that no single approach results in a good performance and that a more nuanced ensemble approach is required. The NLD Model seeks a deft balance among maximizing accuracy, recommendation appropriateness, and business value. For this balance, we employ an expanded ensemble method where, for a given customer and period, the NLD model estimates the probability that the customer will go to a particular city. The model then ranks the destination cities based on the likelihood of travel for a given customer in a given period. It incorporates a loss function to penalize mistakes where the weight is proportional to the customer value. Our incorporation of self-competence ensures that the suggestions are reasonable, and the model avoids making a suggestion when it is not sufficiently confident. We used open-source packages, including Libfm,^1^ LightFM,^2^ contextual bandits [14], and Scikit-learn.^3^ We implemented these baseline algorithms in the Surprise library.^4^ This library is open-source, which facilitates the replication of our algorithm by other researchers.

**Fig. 2:**
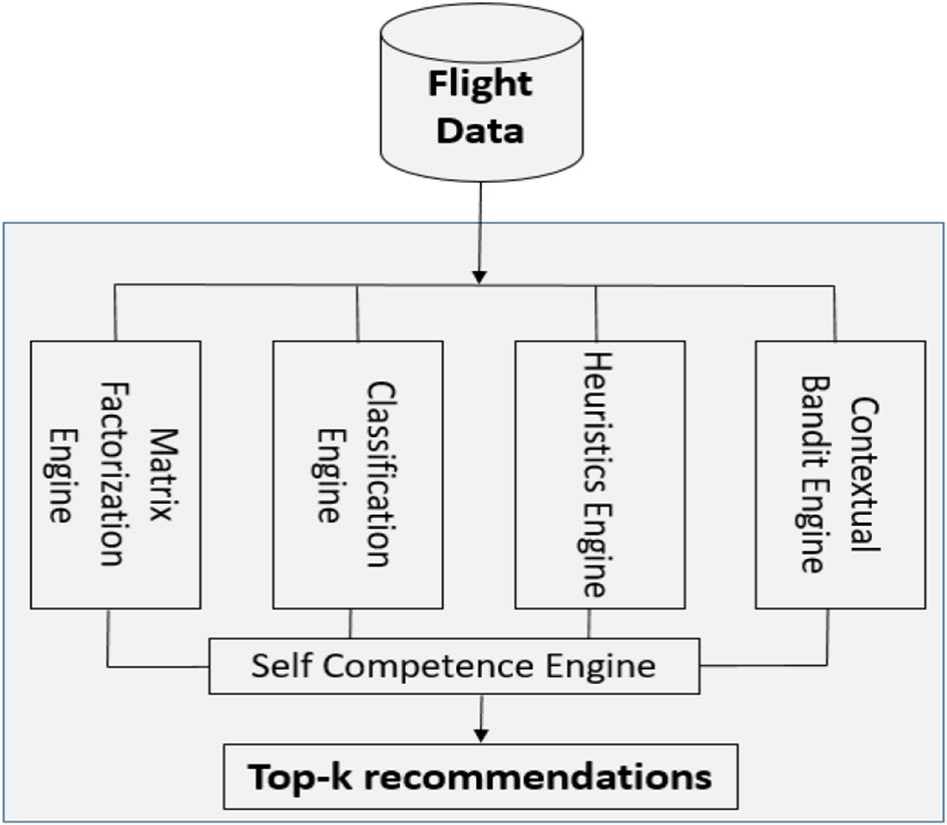
Methodological framework of the NLD model

The NLD Model consists of four integrated classes of algorithms that are assembled in an ensemble framework, which consists of traditional matrix factorization-based recommendation, multi-class classification, rule-based recommenders, and bandit-based recommenders. Into this ensemble, we incorporate a method for calculating the prediction confidence threshold (i.e., self-competence), as shown in Fig. 2.

Our proposed approach differs significantly from prior ensemble-based approaches. For example, the seminal work [30] combines five diverse types of recommenders (SVD, Neighborhood-Based Approaches, Restricted Boltzmann Machines, the Asymmetric Factor Model, and Global Effects). The intuition is that each of these models focuses on different aspects of recommendation, so a blended recommender would outperform any of these individual recommenders. As we show later in our experiments, such a traditional ensemble approach does not work well for the ND problem. A key reason is that these approaches cannot easily incorporate business requirements, such as preferential treatment of certain customers and allowing experts to specify their domain knowledge. Hence, we propose a novel approach with NLD that combines four individual models with diverse and complementary properties. These complementary individual models allow for the incorporation of customer preferences and domain knowledge heuristics. Next, we describe the individual approaches and the rationale for choosing each of them.

Inputs are in three general categories: (a) *customers* (e.g., gender, age, nationality, and membership tier), (b) *information about trips* (e.g., source, destination, booking/flight date, and seating class), and (c) *touristic metadata* about each city, such as geography (e.g., country, weather, and distance), type of tourism served, the best time to visit, and distance from the home airport. The reasons for selecting these four classes of algorithms are:

- *Matrix factorization Engine* matrix factorization (MF) is the dominant recommender system paradigm [39]. We chose the specific hybrid variant because it can handle user and item features [76].
- *Multi-class classifier engine* Classifiers are known to be better able to use features than the recommender system and can address the cold-start issue for new customers [45] and, in our case, very infrequent customers.
- *Rule-based engine* A rule-based model allows domain experts to specify heuristics. This is in line with the emerging field of weak supervision [15, 66], which enables domain experts to specify mostly valid rules [32].
- *Contextual bandits engine* Contextual bandit engines can handle the cold-start problem very well [89], but they are also data-driven and can detect new patterns early [2].

We now discuss each of these ensemble components in more detail.

#### Hybrid matrix factorization engine

**Fig. 3:**
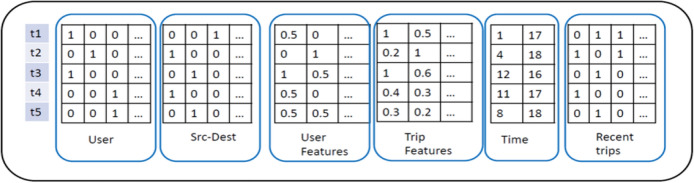
Illustration of the NLD hybrid matrix factorization. Each row/column corresponds to a trip and a real-valued feature, respectively. Src = Starting city. Dest = Destination city

A hybrid matrix factorization engine is a factorization machine that provides a flexible mechanism for incorporating feature engineering into factorization. The generality of factorization machines allows for mimicking and evaluating a variety of matrix factorization algorithms [67]. Specifically, we jointly factorize the user-item, item-feature, and user-feature matrices. The sum of the latent representations of their features represents each user, which enables us to generalize to new users and new source-destinations. For hybrid matrix factorization, we have a set of features encoding customer information using one-hot encoding [51] to denote the various customer features, such as gender, age range, nationality, and airline membership tier. The next set of features identifies the source-destination pair. Then, we incorporate customer features, trip features, time, and recent trips. The NLD model extends MF machines with a weighted variant assigning different weights to different customers [72]. The hybrid matrix factorization feature development is illustrated in Fig. 3. This approach was chosen as it provides an elegant approach to holistically incorporate both customer and trip features. Furthermore, in a number of recommendation-related tasks, matrix factorization-based approaches provide state-of-the-art results [78].

#### Multi-classification engine

We evaluated several classifiers, finding that random forests [8] provided the best results. We also biased the model such that it preferentially focuses on certain relevant customers. Each customer *C*_*i*_ was associated with a weight of *w*_*i*_ based on their importance. Each training example belonging to *C*_*i*_ was assigned the weighted *w*_*i*_. The random forest uses this information when choosing the best split by choosing the benefit in a weighted manner. This approach was chosen to integrate the substantial progress that has been achieved in multi-class classification [73].

#### Rule-based engine

Intuitively, many customers have predictable travel patterns that might not always be identified by a purely data-driven approach. A rule-based approach has several advantages, including being easily interpretable and straightforward for domain experts to specify, and can also help address cold-start issues [45]. We included a rule-based approach [44] as it provides a number of advantages. First, it allows experts to incorporate domain expertise that is not possible in other approaches. Second, recommendations based on rules are easily interpretable for the customer. Finally, the aforementioned two approaches do not work well for a cold-start scenario, such as when a new customer arrives. A rule-based engine addresses these issues [44, 70]. The NLD model considers three rules that cover most of the common travel patterns [63].

- *Contextual rules* One can specify travel patterns in terms of user preferences, trip metadata, and relevant contexts. For example, a customer could be considered an expatriate if her nationality differs from the source city. The customer could be considered interested in skiing if she has visited popular skiing locations during winter. It is also often possible to learn or infer from domain experts’ contextual rules. An example: *RULE 1*: Most expatriates visit their hometown during Christmas.
- *Trend-based rules* Travel patterns can be identified by individual and community trends. For example, an individual trend could be established if the customer attends the next World Cup in soccer as she has done in the past. Community trends are indicators of popular destinations as well. For example, the *Game of Thrones* television show contributed dramatically to the number of visits to film locations.^5^ By analyzing popularity patterns in past trends, the susceptibility of a customer to new trends can be identified.
- *Constraint-based rules* Constraints, such as weather, visa availability, and budget, limit the number of travel patterns. For example, customers might take at most one European trip per year, or customers from certain countries might prefer traveling to countries with visa on arrival, and so on. Often, these categories of constraints are identified from conversations with domain experts.

#### Contextual bandits engine

Our NLD model leverages an exploration/exploitation booking suggestion formulated as a contextual bandit problem [43]. Intuitively, our approach proceeds in three phases. In the first phase, the algorithm is provided with a customer and a set of arms. Each arm corresponds to various destinations represented as a feature vector, blending the context of user and destination. In the second phase, the algorithm chooses an arm by invoking a policy on the feature vector. The selection of an arm results in a reward that is dependent on the customer and destination, such as the ticket class chosen. Finally, based on the reward/penalty, the algorithm improves its arm selection strategy. The aim of contextual bandit-learning algorithms, such as LinUCB [43], is to minimize the regret between the strategy used and the optimal strategy. Unlike the aforementioned approaches, contextual bandits [84] are inherently dynamic and can quickly adapt to changing preferences without the need for retraining.

#### Self-competence engine

Since there is a monetary cost for incorrect ND predictions, it is important to make predictions only when the NLD model is fairly certain. Therefore, the NLD model can choose to abstain if it is not confident. This situation is analogous to a human expert saying, “I do not know,” which is often preferable to making incorrect guesses. A natural approach is to assign a threshold and make the prediction only if the confidence level is above this threshold. This can be done by calibrating the classifiers [86] and then applying the threshold to the output.

However, we advocate for a two-step, cost-aware approach. We first train the recommender/classifier engines on the data using the traditional approach. The NLD model then assumes the availability of misclassification costs for proposing an incorrect destination. Our objective is to identify a cost-aware *abstention range*, a range with a lower bound (20%) and an upper bound (75%), within which no classification decisions are made and with the bounds calculated empirically. Next, we adopt the algorithm to plot the cost curve, showing the misclassification cost against the ratio of misclassification due to the abstention window. Once the abstention window is obtained, it is applied to the calibrated classifier/recommender, and a prediction is only made if the calibrated probability is above the abstention window. This approach of self-competence learning of the classifier and making a prediction in a cost-aware manner results in a better performance than a naïve recommender. This self-competence is a novel aspect of the NLD model from prior work.

## Analysis and results

### Experimental setup

We split the historical dataset into training (60%), validation (10%), and testing (30%), respectively, for the dataset of approximately 27 million bookings. We used stratified sampling so that each partition contained similar trips in terms of all attributes. All of our experiments were repeated ten times on different stratified samples.

### Performance metrics and evaluation

**Fig. 4:**
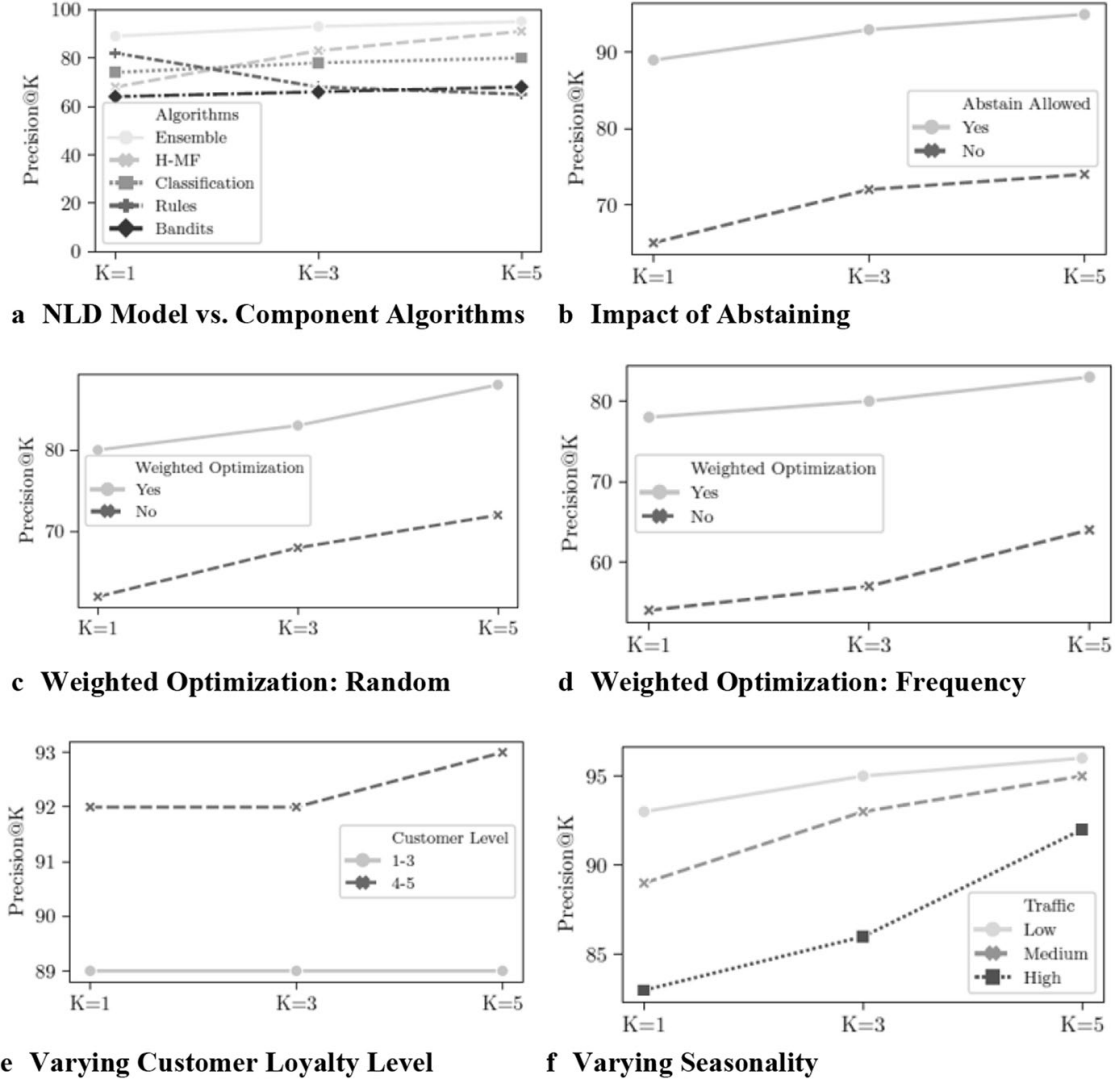
ND algorithmic performance results using historical data. **a** NLD model outperforms components. **b** Abstaining allows the NLD model to obtain increased precision. **c** Weighted optimization improved precision. **d** Weighted optimization improved precision. **e** NLD model performs well at all customer levels. **f** NLD model performs well at all seasonality levels

We optimized for a weighted variant where the misclassification cost is proportional to the distance between the two cities—a proxy for the ticket price. Prior work [52] has noted that the recommendations that are most accurate according to the standard metrics are sometimes not the most useful to customers. Therefore, we moved beyond the conventional accuracy metrics and their associated experimental methodologies and used P@K as the accuracy metric. We used K values of one, three, and five, as most promotional emails from the airline marketing department recommend between one and five locations. This metric allowed us to optimize our ensemble approach and its component algorithms uniformly. It also lends itself to our other solution techniques of calibration and self-competence. We present our results in Fig. 4 and, for readability, Table 2.

**Table 2:** ND algorithmic performance results using historical data

Evaluated component	ND algorithmic performance		
	K@1	K@3	K@5
Ensemble	89	93	95
* H-MF*	68	83	91
* Classification*	74	78	80
* Rules*	82	68	65
* Bandits*	64	66	68
Performance when not abstaining	65	72	74
Weighted optimization (Random)	80	83	88
Weighted optimization (Frequency)	78	80	83
Varying customer loyalty level			
1–3	89	89	89
4–5	92	92	93
Varying seasonality			
Low	93	95	96
Medium	89	93	95
High	83	86	92

#### Comparing NLD model with its components

Figure 4a shows that the NLD model blending multiple algorithms provides excellent results, even for P@1. The Hybrid Matrix Factorization approach is ineffective for small values of K, but it becomes much better for larger values of K. This is acceptable in our context, given the number of locations in most promotional emails. The rule-based approach, for which the rules were mined and verified manually, is effective, as most customers have predictable travel patterns. Once these patterns are exhausted, the performance drops steeply. The NLD model ensures that the overall performance is superior to individual methods via the selection of the destination with the highest predictive value.

#### Impact of self-competence and abstain option

We used a bounded-abstention approach through which the NLD model could abstain from, at most, 10% of the predictions. In practice, at least one algorithm was able to predict the ND for each customer. Figure 4b shows that enabling the abstain option has a nontrivial impact on the performance by improving P@1 by almost 20%.

#### Impact of preferential recommendation

We next evaluate the NLD model’s ability to make recommendations that are business-value aware. Specifically, we assume that some customers are more important than others. Hence, we would like NLD to predict the ND with higher accuracy for these preferential customers. Figures 4c and d show the results. To evaluate the robustness of NLD, we consider and evaluate two ways in which the preferred customers were identified. In the first experiment, we randomly identified 25% of the customers as preferred customers. Since they were randomly chosen, they have very different demographic and behavioral patterns, and this operates as a stress test for NLD. Figure 4c shows that even for this adverse scenario, NLD can predict ND with high accuracy. Figure 4d shows the performance of the scenario where the top 25% of customers in terms of travel frequency are treated as preferred customers, and this is a much more realistic option that is commonly used by the airline. However, this is also a challenging case, as these frequent travelers often have diverse travel patterns, and predicting their ND is tricky. However, the NLD model was able to provide excellent performance results.

#### Varying customer tier level and seasonality

Figure 4e shows the results of grouping customers based on their membership tier, with values varying between one (lowest) to five (highest). We can see that the NLD model gives good accuracy for all groups, with a slight preference for the higher tier. This bias is rational as the NLD model is cost-aware, so making mistakes for these frequent customers results in a higher penalty. Figure 4f shows the result when we group travelers based on seasonality. We considered the aggregate number of travels for each month and partitioned them into three groups: *low*, *medium*, and *high* frequency. For example, December and part of the summer have a lot of traffic, corresponding to the peak travel season. The NLD model has excellent performance when there is limited travel. This high performance is often the case when the flights have low occupancy, and ND could be used to nudge people to travel more. The NLD model performance drops a bit for the peak season due to diverse travel patterns. Nevertheless, P@5 is more than 90%.

#### Varying ensemble models

Figure 4a shows that our novel ensemble approach out-performs each of the individual components. In this experiment, we demonstrate that our proposed approach also out-performs other ensemble approaches. We consider four representative approaches. Ensemble-CF is based on the ensemble algorithm from [30], combining five collaborative filter-based approaches. Ensemble-Tourism is an approach customized for the tourism domain proposed by [57]. We also evaluated the NLD model against two ensemble approaches widely used in multi-class classification, RandomForest and XGBoost. RandomForest [8] is a widely used ensemble approach that constructs multiple decision trees and then combines the predictions of each of these decision trees to output an overall recommendation. RandomForest has the appealing property of avoiding overfitting. Our final algorithm for comparison is XGBoost [13] that implements the state-of-the-art ensemble approach based on gradient boosting.

The result of the experiment can be seen in Table 3. Not surprisingly, our NLD approach out-performs other ensemble-based approaches. This is a testament to our design choice of selective representative recommender algorithms for individual components. Our approach outperforms even the ensemble technique that was custom designed for the tourism domain (e.g., Ensemble-Tourism). The traditional ensemble-based approaches, such as RandomForest and XGBoost, provide the least accuracy, as the other ensemble approaches are more geared toward recommendations. Nevertheless, we incorporate the multi-class classifier as part of NLD’s ensemble, benefiting from their performance.

**Table 3:** ND algorithmic performance results for varying ensemble approaches

Evaluated approach	ND algorithmic performance		
	K@1	K@3	K@5
Ensemble- NLD	89	93	95
Ensemble-CF	78	81	83
Ensemble-tourism	80	81	85
Random forest	63	67	72
XGBoost	65	68	75

As shown, NLD outperforms the baseline by 9% for K@1, 12 for K@3, and 10 for K@5

### Online evaluation

**Fig. 5:**
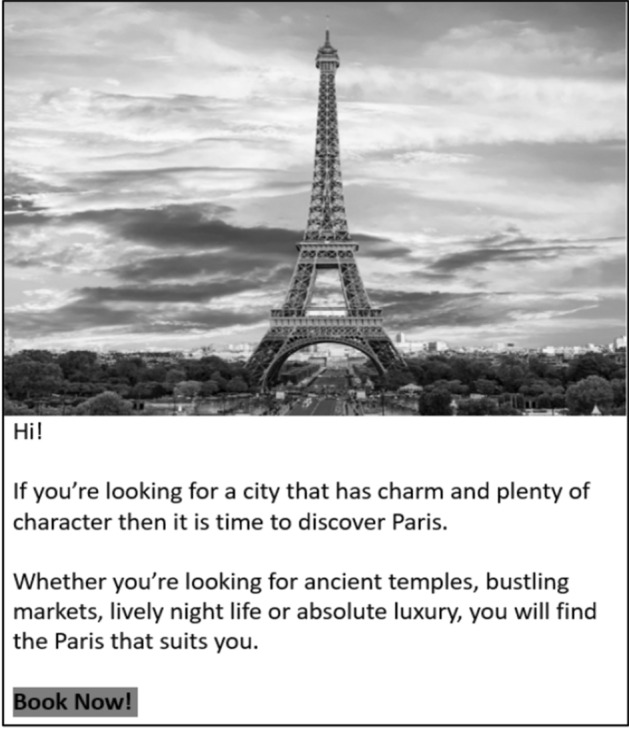
Paris marketing message sent to the test customers. Altered to remove branding

**Fig. 6:**
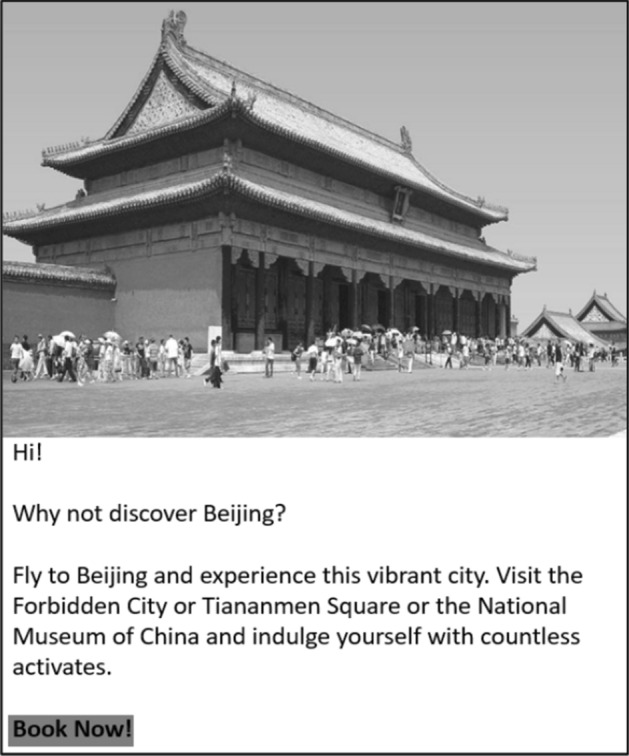
Beijing marketing messages sent to the test customers. Altered to remove branding

Given the excellent performance of the NLD model using historical data, we were optimistic about implementing the model with real customers in a user study to evaluate our model [82]. In cooperation with the airline company, this involved an experiment with 35,000 actual customers (95% test, 5% control). The test customers were targeted by a promotional message for customer enticement of the destinations recommended by our algorithm, and the control group was targeted to a random destination. The test group was sent marketing messages crafted by the airline company marketing department for each destination, as presented in Figs. 5 and 6.

The control group was sent an email message to a random destination that the NLD model did not predict for these customers. Each marketing message contained a recommendation for a selected destination and offered the customer an incentive. The incentive offered was bonus miles for a flight booking to one of the destinations within the offer window.

In our user study, we encountered a challenge that was seldom reported by prior recommender system research. Due to organizational operational constraints, we were given a short booking window of 2 weeks, which is the period when the customer had to book the flight to receive the booking bonus. The additional requirement of a short booking window made the problem much more challenging, as some of these customers still might have traveled to these cities but booked their tickets during a different time window.

The promotional marketing window ran for two weeks. During this user testing evaluation, the NLD model resulted in a prediction accuracy of approximately 51%, as measured by customers booking the predicted destination. Results showed no increase in bookings of the test groups compared to the control group, and there was no change in the booking window. Although the results for the actual user evaluation were disappointing, the results from the combined historical and user testing findings offer impactful insights for researchers in the recommendation domain that are interested in actually deploying their algorithms. We will now discuss these insights.

## Discussion and implications

In this research, we make the following contributions:

- *ND problem introduction* We introduce and study the problem of ND recommendation in the context of the airline industry. Although aspects have been studied in prior research, we confront a host of issues simultaneously.
- *Identification of algorithmic and operational challenges* We identify several algorithmic and operational challenges that make the ND problem formidable, most notably the mixed prediction-recommendation and the incorporation of business value into the evaluation of the model. We present these in a coherent framework.
- *Model development* We develop the NLD model that leverages an ensemble variety of algorithms tailored for ND prediction, and we incorporate the concept of self-competence so that the recommendations have a degree of validity to the actual customer.
- *NLD model development* We evaluate NLD on a real-world historical customer relationship management (CRM) dataset from one of the largest airline companies in the world using standard and business metrics.
- *Evaluation with historical data and real users* We then put the NLD model to the test with real users in an optional setting with a real company’s customers.

We build on prior work in recommendation systems and then expand on this work given that the recommender cannot deal with prediction accuracy as exclusively as usual but must find a balance between prediction accuracy and business value. Specifically, we devise a novel recommendation algorithm for ND in the NLD model, with aspects of (a) temporal and (b) geographic distance between bookings and destinations, as well as incorporate the notions of (c) self-competence and (d) customer-specific weights toward improving business performance. The overall combined accuracy is quite good, even under the exacting conditions of the ND context, with an accuracy of nearly 90% using historical data.

However, the accuracy result of the user study is approximately 50%, which is substantially lower than the results based solely on historical data. So, using historical data does not represent the true predictive accuracy of the model. There are several possible reasons, including the most likely that underlying tastes of the customer population may be in flux and not reflected in historical data [18]. Additionally, there was no statistical difference from the control group. Our premise is that the serendipitous [50] nature of the novel destinations was enticing, which may have induced customers to book at higher rates than the ones suggested for the customers strictly on the predictive aspects. This finding shows that recommendation approaches should be evaluated with real people using real business metrics in a real business context. Again, the aspects of continually changing customer travel taste and serendipitous [50] information encountering may cause a large percentage of travel destinations to be in flux.

The two experiments, however, do expose several interesting aspects of the NLD model. Our evaluations show the relative performance of each of the four adapted algorithms and the combinations of them, with the ensemble approach outperforming the component algorithms. The NLD model also incorporates the novel aspect of self-competence to avoid embarrassingly incorrect suggestions and wasted marketing impressions. This aspect of self-competence greatly improved algorithmic precision. The model is also successful with the ability to optimize the model for specific subgroups of customers. Finally, we present both the historical evaluation of the NLD model and an aspect that is rarely reported in algorithmic research: implementation with real users.

### Algorithmic and empirical contributions

The ND task that we investigated has a host of inherent challenges that the NLD model had to account for in deriving suggestions for the customers. The combined set of these constraints made the ND issue very challenging. Table 4 presents the challenging aspects of the ND problem (corresponding to the ones shown in Table 1) and how the NLD model addresses these challenges.

**Table 4:** Constraints and challenges inherent in ND and the NLD response to address

Constraints	ND challenge	NLD model response
Goal	Mixing of prediction and recommendation	Developed an ensemble approach with some engines weighted toward prediction (e.g., matrix factorization) and others tailored for recommendation (e.g., collaborative filtering)
Interplay of constraints (e.g., timing, location, cost, external events, preferences, revenue, booking window, customer value)	Presence of multiple restraining attributes interacting in determining the ND booking decision	Conducted pilot testing for model refinement; employed feature-based matrix factorization; leveraged rules from domain experts
Business value	Critical aspect of the ND problem	Employed rule-based engine; ranked customers by value; incorporated self-competence to avoid embarrassing mistakes
Data sparsity	Critical aspect of the ND problem	Leveraged collaborative filtering and rule-based engines
Cold start	Critical aspect of the ND problem	Leveraged collaborative filtering and bandit-based engines
Changing characteristics of customers and/or the environment	To be implemented on actual customers of a major international airline	Evaluated on both historical dataset and with real customers
Total set of constraints	ND issue is a complex real-world challenge faced by the airline and similar businesses	Incorporated 4-engine ensemble model with self-competence; used absenting window; ranked customers by value; leveraged rules from domain experts

### Practical contributions

Our work also has several practical implications for the design of algorithmic recommendations within the actual business setting to solve practical problems in a business-aware manner, which are:

- *Model for mixed prediction– recommendation* We introduce an NLD model that is specifically tailored to the airline industry ND issue, specifically considering a mix of prediction and recommendation. We show that business objectives can be integrated into recommender systems by features such as the weighting of customers and self-competence.
- *Evaluation of model with real consumers* The empirical results clearly show that, in a context such as flight booking prediction, one cannot rely solely on historical data. These hybrid recommendation contexts require validation with actual users, rather than relying on exclusively historical information, to get an accurate measure of the algorithmic effectiveness. Given the disparity between results using historical data and those with real customers, it indicates that customer preferences may be in continual flux, requiring an up-to-the-minute tuning of models using customer data.
- *Models must incorporate business objectives* Recommendation approaches must be thoroughly integrated with the overall marketing and customer relations efforts to be effective in achieving the overall business objectives. A challenge in our evaluation was the narrow booking window and the effect of the marketing messages on customer behavior.

### Theoretical implications

Once actual customers are introduced into the scenario, rather than relying on historical CRM data, we found that the accuracy of the NLD model was not as effective at predicting customer booking behavior. For the application of recommender systems in real-world situations, deployed recommendation systems must take into account the unique and possibly changing situations of individual customers. As one possible explanation for this, requiring both algorithmic and customer future research may be expressed in the Individual Behavior Framework (IBF) [19], a psychological theory that posits that individual behavior is expressed via a formula, *B* = *F*(*P, E*), where *B* is a behavior, *F* is some behavior function, *P* is a person, and *E* is the environment around the person.

Most recommendation systems, relying solely on historical data, do not take into account the changing elements [69] of IBF, the person, and the environment. The problem may be that historical information is not the right kind of information or that the medium by which the product information is delivered does not meet the person’s expectations [18, 81]. Also, customer information is constrained by various personal preferences [35] such as holiday or work, expensive or not expensive, etc. This information is private and typically hidden from the airline. An implication is that the airline company should tease out more information from their customers as the typical CRM data is not enough to predict ND bookings. Regardless, our research clearly shows that recommendation algorithms need to account for these changes at the individual level.

## Limitations, strengths, and future work

The limitations of this research also identify areas for future research. First, we address one domain, namely airline bookings. Future research in other areas is needed, but the findings of this research are exciting as a foundation for fruitful studies in the future. A related area for prospective studies lies in the examination of novel ways to expand the typical customer relationship data set to other aspects of flights, evaluating whether these factors play into the context of bookings. The NLD model might perform well in actual user studies if these customer evaluations are targeted to specific segments using this external data [58, 59]. Future research can incorporate and measure the effect marketing messages have on recommender systems in enticing customers to accept a suggestion [81], which the NLD model did not. Another area for future research could include a comprehensive evaluation of the incentives for booking and customer personality types. For example, Knijnenburg and fellow researchers [37] observe that perceptions of recommendation quality and/or variety are important mediators in predicting the effects of objective systems on the three components of user experience: process (e.g., perceived effort, difficulty), system (e.g., perceived system effectiveness), and outcome (e.g., choice satisfaction). We compared our model to the standard baseline ensemble approach, with the proposed model outperforming the state-of-the-art baseline.

## Conclusion

To address the challenges of the ND prediction, we present an NLD ensemble model with the notion of self-competence to provide meaningful suggestions for customer flight bookings. The NLD model balances the need for maximizing accuracy and business value. Our evaluation using a large real-world dataset and a study involving real customers shows promising results and provides insights into businesses that desire to leverage recommender systems in the real world as the findings have implications in a variety of areas. We suggest that, in these contexts, organizations should not rely solely on the historical customer relations data that is typically available to travel companies. Customers do not adhere to the results of the algorithms, at least in the airline domain.

## Footnotes

1 http://libfm.org/

2 https://github.com/lyst/lightfm

3 https://scikit-learn.org.

4 http://surpriselib.com/

5 https://www.fluidandform.com/6-real-life-game-of-thrones-locations-you-can-visit/
